# Material Design and Performance Evaluation of Foam Concrete for Digital Fabrication

**DOI:** 10.3390/ma12152433

**Published:** 2019-07-30

**Authors:** Viacheslav Markin, Venkatesh Naidu Nerella, Christof Schröfl, Gyunay Guseynova, Viktor Mechtcherine

**Affiliations:** Institute for Construction Materials, Technische Universität Dresden, 01602 Dresden, Germany

**Keywords:** digital fabrication, 3D printing, foam concrete, mixture design, material testing

## Abstract

Three-dimensional (3D) printing with foam concrete, which is known for its distinct physical and mechanical properties, has not yet been purposefully investigated. The article at hand presents a methodological approach for the mixture design of 3D-printable foam concretes and a systematic investigation of the potential application of this type of material in digital construction. Three different foam concrete compositions with water-to-binder ratios between 0.33–0.36 and densities of 1100 to 1580 kg/m^3^ in the fresh state were produced with a prefoaming technique using a protein-based foaming agent. Based on the fresh-state tests, including 3D printing as such, an optimum composition was identified, and its compressive and flexural strengths were characterized. The printable foam concrete showed low thermal conductivity and relatively high compressive strengths of above 10 MPa; therefore, it fulfilled the requirements for building materials used for load-bearing wall elements in multi-story houses. Thus, it is suitable for 3D-printing applications, while fulfilling both load-carrying and insulating functions.

## 1. Introduction

Foam concrete (FC) is a lightweight cementitious material with a cellular structure produced by incorporating air voids into mortar or cement paste. It can be designed to have a density in the range of 200 to 1900 kg/m^3^. Foam concrete of density lower than 400 kg/m^3^ is used primarily as a filling or insulating material [1,2,3]. Due to the technical and engineering unfamiliarity of most practitioners and the perceived difficulty in achieving sufficiently high strength, in the past few decades, foam concrete has been largely disregarded for use in structural applications. In most instances, foam concrete was used to fill voids, function as thermal insulation, and act as an acoustic damper. Advancements in chemical and mechanical foaming techniques, concrete admixtures, and other additives significantly improved the stability and mechanical properties of foam concrete. Currently, the potential of this material for structural applications is well recognized, and numerous research projects have been focusing on enhancing foam concrete properties, particularly in respect of its mechanical load-bearing performance [2,4,5]. 

Groups working with foresight on digital fabrication have identified the future need for sustainable construction materials that are economically efficient and environmentally friendly [6]. It is expected that when preliminary studies and descriptions of the fundamental principles of digital fabrication with cementitious materials are completed, a further step would be the rethinking of the technology, including the reduction of material outlays and environmental impact. Foam concrete has a low specific weight, which reduces dead loads and thus enables lessening the dimensions of foundations and amount of reinforcement. Furthermore, the low thermal conductivity of the foam concrete makes it possible to decrease the use of further insulation materials, which are mostly based on petrochemical polymers with a high CO_2_ footprint and very limited recyclability. In contrast to such materials, foam concrete is made of mineral constituents with only minor contents of chemical admixtures [7]. Additionally, since the application of extra insulation panels might be no longer required, significant cuts of energy consumption and time for transport and mounting can be expected along with the noise reduction on the construction site. To summarize, foam concrete is recognized as a versatile construction material that is environmentally friendly and technically efficient. 

The Concrete on-site 3D-Prining (CONPrint3D) concept developed at the Technische Universität Dresden facilitates implementation of the advantages of the additive technology in the construction industry [8]. In contrast to the concepts advancing the printing of integrated formwork, CONPrint3D emphasizes the reduction of secondary steps such as the filling of printed mold structures [9,10]. This technology enables the printing of walls of high thickness, whose purpose would be to replace masonry work. The application of foam concrete in the framework of the CONPrint3D concept is promising and potentially enables the production of load-bearing walls and structural elements with properties such as superior thermal insulation, sound absorption, and fire resistance [11,12]. The authors expect that the application of different cement-based materials in concrete 3D printing will simplify the formulation of new building standards and change over to the full automation of construction processes. By varying the density and thickness of 3D-printed foam concrete walls, the complete or partial elimination of additional insulation systems would be possible. A further aspect facilitating the application of foam concrete as a material with both insulating and structural functions is the ease of its recycling and disposal. 

The literature contains an example that describes an automated application of foam concrete to vertical surfaces by means of an extrusion-based technique [13]. The authors placed foam concrete onto the bare walls of existing buildings to gain a facade finish that insulates, is recyclable, and is free in design and form. The material that was used possessed apparent shape stability, whereas strength characteristics were not studied. 

Faliano et al. [14,15] described foam concretes with a dry density between 400–800 kg/m^3^ and compressive strength in the range of 1.5 to 9 MPa, and which in addition maintains its dimensional stability after extrusion. The water-to-cement ratio (w/c) was set to 0.3 in all the mixtures. Neither fillers nor aggregates were used. Preformed foam was prepared with a protein-based foaming agent. The research provides a wide range of results related to the influences of curing conditions on tensile and compressive strengths. However, the experimental procedure described did not represent typical 3D-printing procedures by robotic print heads. The material was rather filled into steel formwork and pushed down manually from the formwork in the early stage of hydration. The deposition technique used by Faliano et al. imitated automated extrusion and provided first filling of the material behavior in terms of form stability and green strength development. 

There is no standard way of measuring the properties of buildability. Generally, buildability is evaluated by printing a certain number of layers at a specific rate [16,17,18,19]. At this point, it is hard to estimate the possible buildability of the foam concrete designed by Faliano et al. [11,12], since the resting time of the foam concrete and its rheological characteristics in the fresh state were not specified. The study emphasized the use of viscosity-enhancing agents (VEA) and indicated more need for research on the fresh state behavior of the extrudable foam concrete. The authors presumed the possibility of applying the extrudable foam concrete mixtures of density down to 200 kg/m^3^. Both structural and non-structural applications for the extrudable elements made of foam concrete were denominated as effective and environmentally friendly. One of the suggested applications was to form multilayer insulating panels in situ.

In general, concrete that is suitable for digital construction must be well extrudable and demonstrate adequate buildability. Furthermore, printed layers need to have good interlayer bonds [9,16,20,21]. Finally, the material has to yield appropriate mechanical properties, e.g., compressive strength [9,21,22,23]. Conventional foam concrete features good workability and flowability, which are promising with respect to the process parameters of extrudability and pumpability, as required for 3D printing. In common applications, foam concrete is pumped to a point of placement and, in general, does not need compaction; foam concrete can successfully be pumped over significant distances and heights [1]. Thus, from this perspective, it is suitable for extrusion-based 3D-printing techniques. However, the potential effects of pumping on the foam characteristics have to be taken into account, since they might influence the stability of the mixture and result in changes in its density. 

Another important feature of a printable material is its buildability, which is comprised of the shape stability of the printed layers under their self-weight and the ability to hold further layers with minimum deformation [20]. In other words, the buildability of foam concrete can be described as a combination of self-stability and sufficient stiffness with early setting. Regarding self-stability, foam concrete is usually perceived as a free-flowing, self-compacting material. It is recognized that at lower densities, the flowability decreases due to the reduced self-weight and adhesion between the solid particles and air bubbles [24]. However, previous research on foam concrete demonstrated that a decrease in flowability with respect to common applications such as void filling is often treated as a sign of poor quality or inappropriate mix design [4]. Having 3D printing as the application technology in mind, achieving pumpable and self-stable foam concrete should be possible, but this approach has not been investigated thoroughly to date, and so, further research is needed. 

In studies related to 3D printing with normal-weight concrete, quick setting is usually achieved by using accelerating admixtures or by choosing cements with shorter setting times, i.e., rapid-hardening sulfoaluminate or calcium aluminate cements [6,25]. The same approaches can be followed to achieve the quick setting of foam concrete. However, as reported in [26], the use of setting-accelerating materials in foam concrete does not always have the same effect as in normal-weight concrete. Moreover, they can cause instability and affect the quality of the foam concrete. In some studies, different types of cement characterized by rapid setting were utilized [27,28]. Rapid-hardening Portland cement is often used to reduce the risks of instability and segregation and ensure that foam concrete will develop a strong homogeneous microstructure at a very early stage. It was also observed that the addition of aluminate cement, while shortening setting times, can decrease the compressive strength of foam concrete [29]. Moreover, the special cementitious materials mentioned are relatively expensive, which limits their range of application. 

Another important aspect of printed elements is their interlayer bonding. It strongly influences the mechanical properties, durability, and serviceability of 3D printed structures; see e.g., [30,31,32]. The quality of the interlayer bond depends on numerous factors related to the properties of the fresh concrete and the printing technique, i.e., the time interval between layers, filament shape and size, etc. No literature was found that could help estimate foam concrete’s behavior from this point of view. Regarding foam concrete’s permeability and resistance to aggressive environments, it was proven that its cellular, porous structure does not necessarily make it less resistant to the penetration of moisture in comparison to conventional, dense concrete, since the air voids are not interconnected and appear to act as a buffer preventing capillary suction and other transport processes.

Generally, there are two mechanisms to introduce large volumes of air voids into the mixture: (1) the use of gas-forming chemicals such as aluminum powder, and (2) the use of foaming agents. Addition of the gas-forming agents results in bubble formation through chemical reactions with alkaline hydration products, e.g., calcium hydroxide [33]. This method is used to produce gas concrete, which is also called aerated concrete. As reported by Holt and Raivio [31], aerated concrete produced by adding aluminum powder has some significant drawbacks, such as its relatively high cost as well as its lower strength, higher moisture content, and more pronounced shrinkage when compared to traditional concrete. The properties of aerated concrete can be considerably improved by autoclave high-pressure steam curing. However, such curing would be counterproductive, since the main benefit of the production technique of 3D-concrete printing is the reduction of interim steps such as elaborate casting and curing.

In the alternative approach, foam concrete can be produced either by adding the foaming agent to the cement paste, followed by intense mixing, which is called the mixed foaming method, or by intermixing a separately produced foam into cement paste, which is known as the prefoaming method [1,4]. In contrast to the addition of gas-forming chemicals, the use of foaming agents in producing the foam concrete has a higher potential for application in 3D printing. This is mostly explained by the fresh and hardened properties’ relative ease of adjustment by varying raw materials and chemical admixtures [1,2,7,24,26,34]. 

The mixed foaming method is widely used in the construction industry to produce foam concrete. However, this method is limited according to the use of the synthetic foaming agents and is highly dependent on the mixing device used. In contrast, the prefoaming method enables the defining of the material density by the exact addition of the required amount of the foam to the base mixture. Since the ratio of foam to base material could be greater than 1:1, foam becomes a major influencing factor [35]. The stability of the air voids during pumping and intermixing to the cement-based matrix are essential to ensure the required performance of the foam concrete in the fresh and hardened states. For foam concrete applications, synthetic foaming agents are easier to handle, less susceptible to extreme temperatures, and they can be stored longer. Synthetic foaming agents can be used in both prefoaming and mixed-foaming techniques. Moreover, they are generally less expensive and require considerably less energy to produce high-quality foams [35]. Nevertheless, synthetic surfactants cannot match the performance of protein-based agents due to their larger bubble size and less isolated cells, which result in lower concrete strengths [35,36]. The foams produced with protein-based foaming agents are characterized by the smaller size of air bubbles, higher stability, i.e., lower water drainage, and stronger isolated bubble structure in comparison to foams produced by synthetic foaming agents [1,2]. It was also reported that foam concrete produced with the use of protein-based surfactants has a strength-to-density ratio from 50% to 100% higher compared to foam concrete produced with the use of synthetic foaming agent [35,36]. 

**Figure 1:**
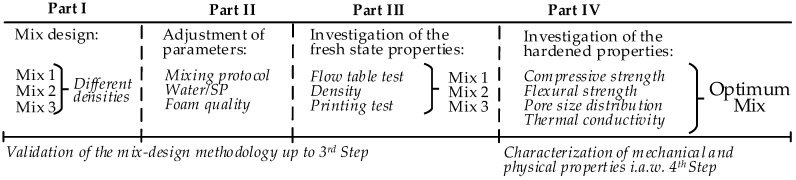
Overview of the experimental program.

Based on the considerations mentioned regarding the performance of two existing surfactants, this study focuses on the prefoaming production technique using a protein-based foaming agent. Figure 1 shows the structure of the experimental part of the presented study. The study at hand is dedicated to achieving a printable foam concrete, which is stable and yields adequate rheological and mechanical properties that are suitable for 3D printing. The constituent materials were chosen purposefully to achieve sufficient cohesiveness and form stability right after the deposition of the material by the print head, as well as adequate long-term mechanical properties for structural applications. Four recipes were prepared. The desired density of the fresh mixes was specified between 1100–1600 kg/m^3^. Finally, the insulating properties of the printable foam concrete were compared to those of normal-weight printable concrete (the reference material is described in [37]). 

## 2. Materials and Methods

### 2.1. Mixture Design Methodology and Experimental Program

**Figure 2:**
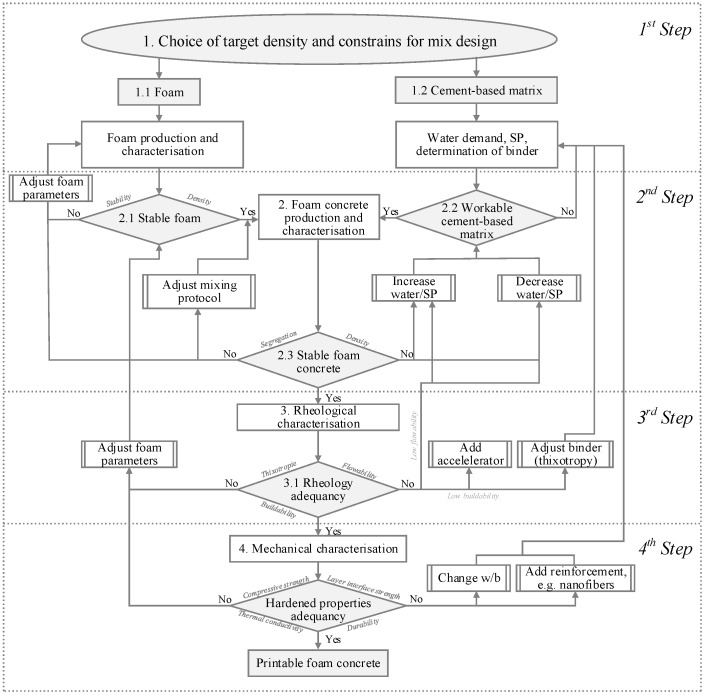
Mixture design approach for printable foam concrete.

The scheme of the mixture design approach as developed in the framework of the research project CONPrint3D-Ultralight is presented in Figure 2. This approach can also be applied to the mixed foaming method. Then, foam characterization is unneeded. The mixture design of a foam concrete using the prefoaming method is divided into two steps, namely establishing the cement-based matrix composition and determining the amount of foam to be added to achieve the desired density. In particular, the overall mixture design approach can be split into four steps, as shown in Figure 2. Iterative optimization is used to achieve satisfying printable foam concrete compositions. 

Firstly, constraints such as the range of the water-to cement ratio (w/c) and cement content must be set according to the intended application. Based on information from the literature, suitable proportions and materials can be identified. Production and characterization of the foam follow. The aim of this step is to obtain sufficiently stable foam that is capable of withstanding the intermixing process. In parallel, the water demand and binder composition of the cement-based matrix, including the superplasticizer (SP) dosage, are determined by iterative testing. Workability was assessed by measuring the spread flow diameter values in accordance with the European standard DIN EN 1015-3:1998, and thus using the so-called Hägermann cone and by applying 15 strokes [38]. In the first run, the goal of this procedure is to produce a cement-based matrix with minimal amounts of water, yet that is still sufficient to plasticize the matrix with the recommended dosage of SP. At the same time, the cement-based matrix must be flowable enough to ensure good incorporation of the foam into the mixture. An excessively stiff cement-based matrix leads to foam breakage or collapse, whereas an overly fluid matrix segregates. In this investigation, the first estimation of water addition was done following a procedure described by Okamura and Ozawa [39]. As a result of the first step, there is a stable foam and an appropriately fluid cement-based matrix.

The third step focuses on verifying the fresh foam concrete’s rheological properties, which have to comply with the 3D-printing process requirements of printability, extrudability, and buildability [39,40,41,42]. The binder composition can be adjusted to achieve the required properties, including the use of further chemical admixtures and further optimization of the foam. 

The last step specifies testing the hardened state properties of foam concrete, such as its compressive and flexural strengths, thermal conductivity, and/or durability. At this stage, the water-to-binder ratio (w/b) may be reduced; alternatively, reinforcement in the form of dispersed nanofibers or microfibers may be introduced [1,3,43]. The approach presented in Figure 2 was used in this study to develop foam concretes with varying densities by the variation of their composition and mixing regimes. Fresh state rheological and hardened state mechanical properties of the mixtures—following the scheme in Figure 1—were tested, and their results are presented in Section 3. 

### 2.2. Determination of the Water Demand

It is essential to specify a suitable water content in the foam concrete. There is no standard procedure, especially when the requirements of printability, pumpability, and buildability must be fulfilled. In the present work, the water demand of the cement-based matrix was determined by Okamura’s and Ozawa’s method [39]. The compositions of powders tested are listed in Table 1.

### 2.3. Raw Materials

**Figure 3:**
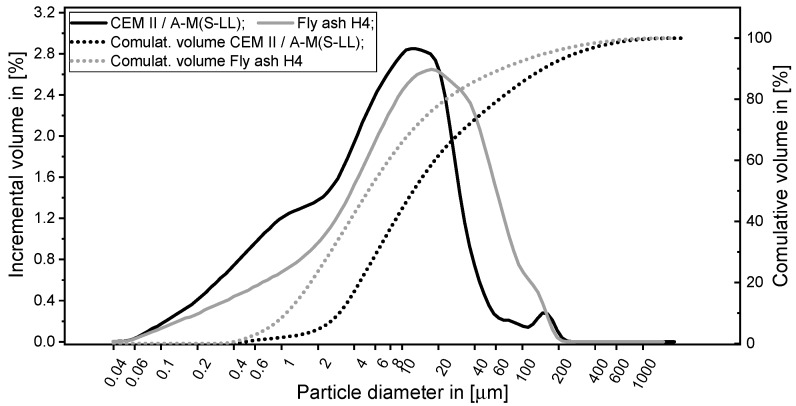
Particle size distribution of the solid constituents.

A type II Portland composite cement CEM II/A-M (S-LL) 52.5 R (OPTERRA Zement GmbH, Werk Karsdorf, Germany) was used. Hard coal fly ash Steament H-4 (STEAG Power Minerals GmbH, Dinslaken, Germany) was chosen as a secondary cementitious material. The chemical composition and measured particle size distribution are provided in Table 2 and Figure 3, respectively. While the chemical composition was taken from the datasheets of the materials’ providers, the particle size distributions were assessed by laser diffraction (LS 13320, Beckman Coulter, Krefeld, Germany). The fly ash complies with DIN EN 450 [44] and is permitted to be used as an additive to concrete according to DIN EN 206-1 [45]. Thus, it was accepted as obtained in the study at hand and not characterized further. Minor constituents are shown in Table 2, whereas no values are provided for the major constituents SiO_2_ and Al_2_O_3_. The implementation of fly ash into concrete composition on the one hand enabled decreasing the water demand of dry constituents while still achieving the targeted rheological behavior; on the other hand, it improved the sustainability of the mixtures. A polycarboxylate ether (PCE)-based SP (MasterGlenium SKY 593, BASF Construction Solutions GmbH, Trostberg, Germany) was used in the cement-based matrix to adjust the workability at reduced water content. The water content in the SP was 77% by mass. The density of the SP equaled 1050 kg/m^3^. A protein-based foaming agent (Oxal PLB6, MC-Bauchemie GmbH & Co. KG, Bottrop, Germany) was utilized for production of the foam. 

### 2.4. Mixing Procedure

In a preliminary stage, three liters of the cement-based matrix paste were produced to assess the water demand using a pan mixer (Hobart NCM20, The Hobart Manufacturing Company Ltd, London, UK, 5 L capacity). Table 3 describes the mixing procedure.

Foam concrete was produced using a conical, multi-rotor colloidal mixer (KNIELE KKM30, Kniele GmbH, Bad Buchau, Germany). For each experiment, 30 L of foam concrete was prepared using the procedure according to Table 4. After the binder matrix was mixed, the separately produced foam was added stepwise: 40%; then, another 40%, and finally, the remaining 20% of the total foam volume. 

### 2.5. 3D Printing Process 

**Figure 4:**
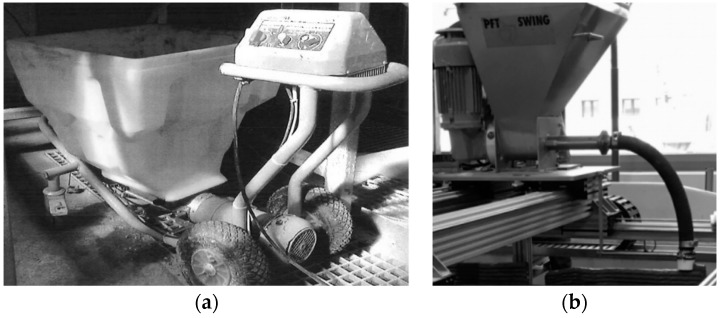
(**a**) The standalone progressive cavity pump (PCP), DUROPACT DP 326S, and (**b**) 3D concrete printing test device (3DPTD).

Extrusion and deposition experiments were conducted using two devices: (a) a standalone progressive cavity pump (PCP1) DURAPACT DP 326S (DURAPACT Gesellschaft für Faserbetontechnologie mbH, Haan, Germany), and (b) the 3D concrete printing test device (3DPTD, a 3D-Printing device for concrete custom developed by TU Dresden, Dresden, Germany) equipped with PCP2; see Figure 4. A 25-mm diameter pipe was used, while its nozzle outlet was positioned manually to deposit concrete layers. In Figure 4b, the nozzle outlet is positioned autonomously with a pre-programmed Lua script, which is a programming language. When using PCP1, the pumping flow rate was set to 10 L/min, and the nozzle outlet had a round cross-section with a diameter of 20 mm. Printing experiments with custom-developed 3DPTD were performed with two different rectangular nozzle geometries of 10 mm by 50 mm and 20 mm by 30 mm to investigate the influence of this parameter on the printability performance of foam concrete. A printing speed of 40 mm/s was selected based on preliminary extrudability investigations. Straight wall specimens with a length of 700 mm were produced with layer-to-layer deposition time intervals of 30 s. To assess the buildability of a mixture composition, a maximum number of layers was deposited, one atop the other, until self-collapse occurred. Additionally, walls made of only three layers were printed and eventually used in the preparation of specimens for mechanical testing. 

### 2.6. Specimen Preparation

Each printed wall was transferred to a climatic chamber at an age of 24 h and cured at a constant temperature of 20 °C, a relative humidity of 65%, and in the absence of wind for 27 days. This procedure on purpose does not accord to DIN EN 12390-2 [46], which prescribes very different curing conditions, namely moist curing. Since no formwork is used in concrete 3D printing and since practicable curing options are very limited due to the particularities of the printing process, the authors decided to use a standard laboratory climate throughout the entire experimental program, including concrete preparation, 3D printing, curing, and testing. Such climatic conditions best represent the perspective exposure of large-scale printed structural elements in the practice of construction. At the age of six days, the walls were saw-cut to make specimens for mechanical testing. The sawing occurred without the addition of water to avoid absorption; then, the specimens were returned to the climate chamber. Cubes with an edge length of 40 mm were prepared for compressive strength testing, whereas the dimensions of specimens for the bending tests varied in a range of 30 to 33-mm width and 50 to 56-mm height, which corresponds to that of three printed layers. Uneven sideward surfaces of the layers were not polished. The length of beam specimens was 160 mm. The loading area was even-tempered with fast-setting gypsum. 

### 2.7. Mechanical Testing 

**Figure 5:**
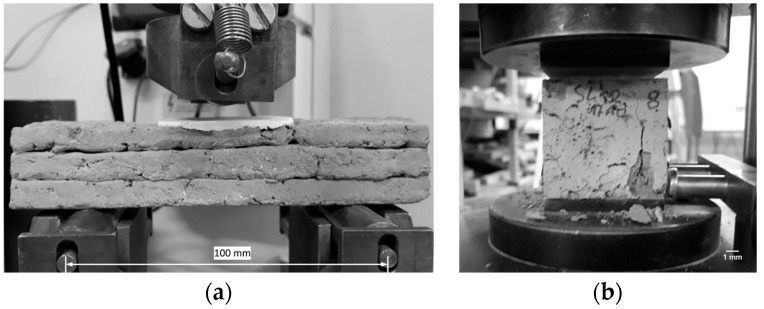
Measurement of the mechanical properties of the printed specimens: (**a**) three-point bending test (Zwick 1445, ZwickRoell GmbH & Co. KG, Ulm, Germany), (**b**) uniaxial compression test (EU20, VEB Werkstoffprüfmaschinen, Leipzig, Germany).

Figure 5 shows the set-ups for the bending and compression tests. The bending tests were performed under traverse displacement control with a displacement rate of 0.5 mm/min. For compressive strength measurements, the loading plates of the test set-up were 40 mm by 40 mm according to the cross-section of the cubes. For each material, no less than three samples were tested. 

### 2.8. Thermal Conductivity Measurements

Specimens of dimensions of 70 × 70 × 20 mm^3^ were saw-cut from the walls printed in the same manner as those for mechanical testing. The insulation properties of the optimum mix composition were measured with a Heat Transfer Analyzer ISOMET 2104 (Applied Precision Ltd, Bratislava, Slovakia). This apparatus applies a dynamic measurement method that enables the reduction of the period of the thermal conductivity measurements to a mere 10 to 16 min. 

### 2.9. Scanning Electron Microscopy and Light Microscopy 

Scanning electron microscopy (SEM) was used to visualize the microstructure of the foam concrete. The SEM unit Quanta 250 FEG (Thermo Fisher Scientific, Waltham, MA, USA) was operated in the so-called “low-vacuum mode”, whereby the non-conductive specimens were imaged as obtained without sputter-coating. 

**Figure 6:**
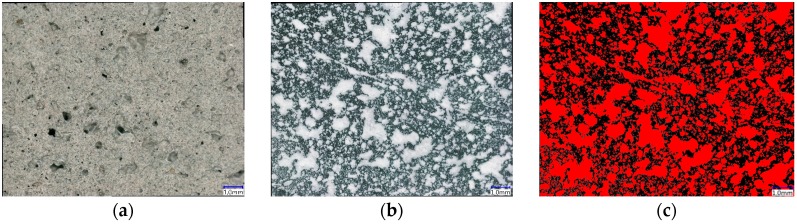
Typical initial image and sequence of processed images of foam concrete: (**a**) polished specimen, (**b**) colored image, (**c**) binary image processed for computational measurements of air-void parameters.

The pore structure of foam concrete consists of gel pores, capillary pores, and entrained and entrapped air voids [3]. Gel and capillary pores were not assessed, because these features of the cement-based matrix were not considered essential in the study at hand. Meanwhile, only entrained and entrapped air voids with diameters larger than 0.01 mm were assessed. The sizes of the air voids in the foam concrete were studied using a digital microscope VHX 6000 (Keyence Deutschland GmbH, Neu-Isenburg, Germany) with a high-resolution image analysis tool. The SEM technique does not allow capturing a large area, but rather requires lengthy imaging sequences and image stitching. Contrarily, the digital light microscope allowed the generation of overview images of the pore-rich microstructure at the most appropriate degree of resolution much easier. The specimens of the thermal conductivity measurements were used further for the porosity measurements. They were processed in three steps: (1) polishing the surface to be observed with sandpapers of different grading, (2) coloring the smoothened surface with a black felt-tip pen, and 3) filling the broached pores with a contrasting color powder (white BaSO_4_). This part of the sample preparation is in accordance with DIN EN 480-11:2005 [47]. An area of 1905.0 mm² was considered for evaluation. After the pores were filled and the contrast between the pores and the remaining surface was archived, a binary image consisting of two (random) colors was generated. Figure 6 shows a typical image processing sequence.

## 3. Results and discussion

### 3.1. Determination of Water Demand of the Powders

The water demand $\beta_{p}$ was obtained for a pre-selected powder mixture by extrapolating relative slump values $\Gamma_{p}$ (Equation (1)) linearly from the measurements for various volumetric water-to-powder ratios to the zero slump. At this state, the amount of water in the paste is assumed to be just enough to wet the surfaces of all the particles and fill all the voids in the system (saturation point). Note that no admixtures are used in such a characteristic stage of the study.

(1) $$\Gamma_{p}={\left[\frac{(D_{1}+D_{2})/2}{D_{0}}\right]}^{2}-1,$$

where $D_{1}$ and $D_{2}$ are the spread diameters obtained in slump-flow tests (measured in two directions), and $D_{0}$ is the base diameter of the conical frustum used in the test (here: 100 mm).

**Figure 7:**
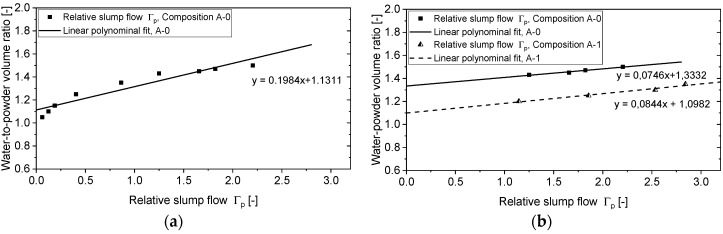
Results of relative slump values for the binder pastes: (**a**) all values for the composition A-0, and (**b**) results obtained in the range Γ_p_ >1 for the compositions A-0 and A-1.

Figure 7a shows all the results of $\Gamma_{p}$ for composition A-0, including those smaller than one, whereby a non-linear relationship between water-to-powder ratio and $\Gamma_{p}$ can be seen for small values of the latter. When all the measurements of $\Gamma_{p}$ are considered, the resulting $\beta_{p}$ would be 1.13, which might lead to a very low w/c and an insufficiency of water in the mixture. According to the existing guidelines [48,49], only the region $\Gamma_{p}$ > 1 is taken into account for linear extrapolation to obtain. Figure 7b illustrates this case. The water demand $\beta_{p}$ obtained in this way for the compositions A-0 and A-1 equals 1.33 and 1.10, respectively. 

The water demand of self-compacting concrete following Okamura’s procedure is usually set at 80% to 90% of the experimentally determined $\beta_{p}$ value, with the transition from paste to mortar or concrete. Various effects of the additional mineral ingredients in concrete as compared to the mortar are considered in the form of this scaling factor [48], but first of all, it considers the effect of the superplasticizers (SP) added for the production of mortar and concrete, in contrast to the paste, which was tested without the addition of SP. In the present study, such a reduction factor was introduced individually due to the characteristics of the prefabricated foam. Taking into account the instability of the foam over time, e.g., 30 g of water per liter of foam drained in a time period of 35 min, and the observation that a certain volume of the foam collapses during intermixing into the cement paste, the experimentally determined value of $\beta_{p}$ was reduced by 25%.

### 3.2. Mixture Design

For the first foam concrete experiments, composition A-1, consisting of cement and fly ash, was chosen. The foam volume was calculated according to the method described in [36], which does not take into account the amount of water in the foam; instead, it includes only the water in the cement-based matrix. Contrarily, the w/c of foam concrete is in fact increased by the water content in the foam. Thus, in the study at hand, the whole amounts of water both in the foam and in the HRWRA were subtracted from the total water content that had been determined by Okamura’s method. Three mixture compositions with different densities were designed; see Table 5.

### 3.3. Properties of Foam Concrete in Its Fresh State 

#### 3.3.1. Flow Table Test

Table 6 summarizes the properties of the foam concrete mixtures. All three compositions exhibited similar spread values, which stem from the comparable proportions of constituents and similar water-to-binder ratios. Upon the addition of foam, the spread values after strokes did not change significantly. The differences in the density of the mixtures could be expected to influence the spread values when considering related differences in gravitational forces. However, while the density increased from M-1 to M-2 to M-3, the content of the cement paste increased as well, hence leading to higher static yield stress and more pronounced structural build-up at early ages. The increase in yield stress seems to counteract the increase in gravitational force, so that all the tested mixtures had similar spread values.

**Figure 8:**
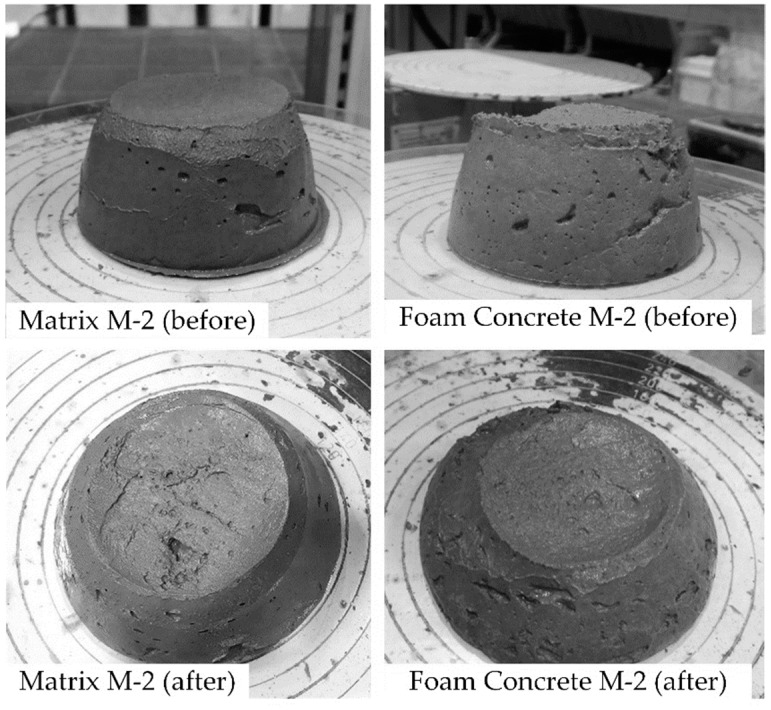
Flow spread of the cement-based matrix and the corresponding foam concrete composition M-2 before and after strokes.

The spread diameters of all the mixtures comply satisfactorily with the requirement for printable concrete, which was shown to be between 120–140 mm [11]. Figure 8 shows exemplarily spread images of the composition M-2 with and without foam addition.

#### 3.3.2. Verification of the Density 

A common challenge in foam concrete production is the instability of its pore system and the resulting deviation from the targeted density, which is hereafter termed density discrepancy. The risk of such discrepancy is increased in the case of printable foam concretes due to their relatively stiff cement-based matrix. Therefore, the mixture design approach of the study at hand incorporates a step for the revision of the concrete composition based on this discrepancy in the density achieved, as compared to the initial target density. Table 7 presents the results of the density measurements.

Mixture M-1 had an initial target density and a finally measured fresh mixture density of 1100 kg/m^3^ and 1180 kg/m^3^, respectively. Similarly, M-3 had a fresh density of 1430 kg/m^3^, which differed markedly from the target value of 1580 kg/m^3^. Interestingly, the density discrepancy of M-2 was almost negligible in comparison to that of the other mixtures. The results presented in [36] indicate that the instability of the foam concrete may be caused by a range of factors. Generally, any deviation from the target density can be attributed to the stability of the foam (collapse/drainage), mixing time, mixing speed, and the design of the mixer used. Prospective rheological and microstructural investigations should contribute to the remedy of the instability of the printable foam concrete observed in this study. It was clearly observed that the foam added could either increase its volume during the agitation, or partially collapse, which are both processes that significantly alter the density. 

The density of the foam concretes changes over time due to drying. The kinetics of this process and the resulting dry density depend on ambient temperature and humidity as well as on maturity, fresh density, and w/c of the concrete, and on the area of the surface exposed to evaporation. The drying weight loss varied with the density; see Table 8. While the printable foam concrete composition with a fresh density of 1580 kg/m^3^ (M-3) had a weight loss upon drying of just about 9%, higher amounts of foam added gave rise to pronouncedly higher drying losses of approximately 18% (M-1 and M-2). 

#### 3.3.3. Extrudability and Printing Test

**Figure 9:**
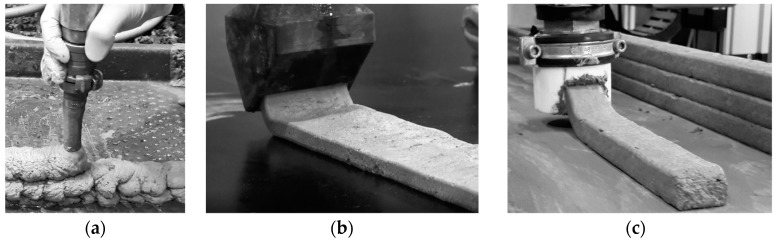
Extrusion experiments with designed foam concrete: (**a**) composition M-1, extrusion with a standalone progressive cavity pump (PCP) DURAPACT DP 326S, (**b**) composition M-2 and (**c**) composition M-3, dispositioning with 3DPTD equipped with a PCP.

All the mixtures could be extruded and deposited during the first 20 min after production without any visible deformations upon deposition. During the preliminary investigation, M-1 layers were extruded and deposited manually; see Figure 9a. Imperfections in the manual guidance of the extrusion nozzle made a quantitative evaluation of the buildability of M-1 impossible. Despite this, the first trial extrusion of the designed foam concrete verified the appropriateness of the mix design approach for printable foam concrete, as described in Section 3.1. 

The buildability of the M-2 and M-3 foam concretes was evaluated by printing a maximum number of layers until collapse of the wall specimens. The maximum heights and number of layers of printed walls with M-2 and M-3 equaled 15 cm with 16 layers and 10 cm with six layers, respectively. Note that the geometries of the nozzles varied between M-2 and M-3, and the aspect ratios (height to width) were 0.2 and 0.7, respectively. The edges of all the layers retained the rectangular default shape imposed by the nozzle, and no significant deficiencies were noted on the surfaces of the deposited layers. In the observation period of 24 h after printing, no visible cracks or other signs for early age degradation were found. 

**Figure 10:**
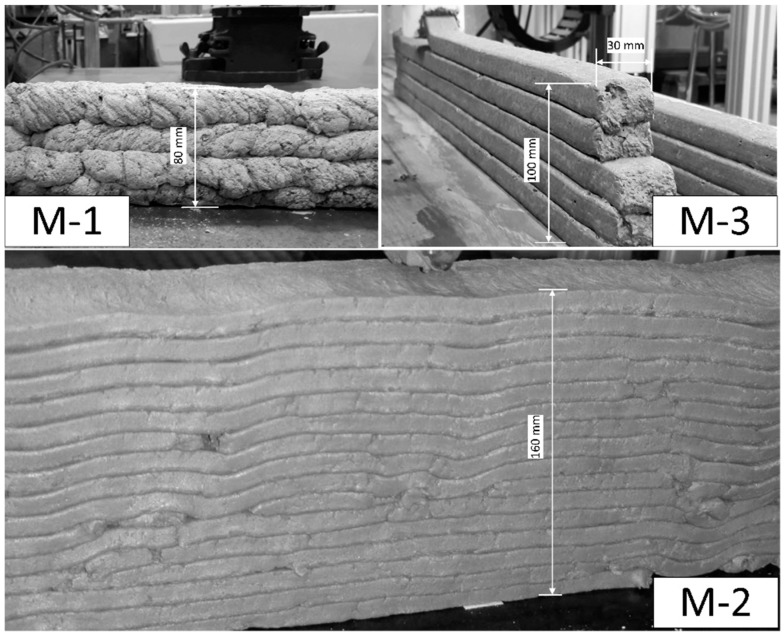
Extruded wall samples. M1 was extruded manually.

Figure 10 shows the wall specimen of the composition M-2 with its discontinuities in the printed layers. This phenomenon was not observed for composition M-3, which had a higher density. It is worth noting that in comparison to mixture M-3 with an air content of 20%, mixture M-2 had an air content of 40%. This observation leads to the conclusion that the reduction of foam concrete density affected printing characteristics. Previous research on foam concrete rheology shows that as the air content increases, the plastic viscosity also tends to increase. This underlines the role of the air content on the ability of foam concrete to flow under an applied shear stress [50]. In addition, due to the higher air content and related lower gravitation force, the feed of material at the inlet of the extruder may not be continuous, which could also have led to the discontinuities in case of M-2. 

Of the three compositions designed, M-2 revealed the lowest discrepancy from the target density; see also Section 3.3.2. In addition, by external pursuit of a continuous material feed, the reasons for certain discontinuities as mentioned above are considered to have been overcome. Thus, M-2 is regarded to be a suitable mixture composition for the printing of formwork-free, load-bearing foam-concrete walls. Its mechanical and physical properties have been evaluated and are presented in the next section.

### 3.4. Properties of Foam Concrete in the Hardened State

#### 3.4.1. Mechanical Properties

Table 8 presents the results of the compressive and bending tests on the hardened mixture M-2. The average values of the compressive and flexural strength of the printed foam concrete at an age of 28 days were 10.4 MPa and 2.12 MPa, respectively. Thus, they are perfectly in the range of the values reported in the respective literature on conventionally cast foam concretes that feature comparable densities [51,52]. Various publications reported on enhancing the mechanical characteristics of foam concrete, pointing to possibilities of increasing its compressive strength, see e.g., [53]. While some measures such as the use of nanoadditives can enhance mechanical performance, it should be kept in mind that the compressive strength of foam concrete will remain strongly linked to its density. With density as the main factor influencing insulating characteristics, the set of hardened properties of foam concrete, i.e., its compressive strength and density, must be well specified, particularly in consideration of target applications. 

#### 3.4.2. Thermal conductivity

Table 9 presents the thermal conductivities of foam concrete M-2 in comparison to conventional concrete and a printable, fine-grained, ordinary-density concrete mixture V-1, which was detailed in [54]. The conventional concrete with a density of 2200 kg/m^3^ and the printed concrete with a density of 2100 kg/m^3^ exhibited similar thermal conductivities of 1.70 W/mK and 1.68 W/mK, respectively. Contrarily, printable foam concrete M-2 with its closed-cell meso structure was found to have a thermal conductivity of 0.24 W/mK, which is approximately 85% lower than that of the reference concretes; see also Section 3.4.3. Furthermore, the thermal conductivity of 3D printed M-2 was similar to ordinary foam concrete, with the same density reported in [33]. 

#### 3.4.3. Microstructure and Porosity

**Figure 11:**
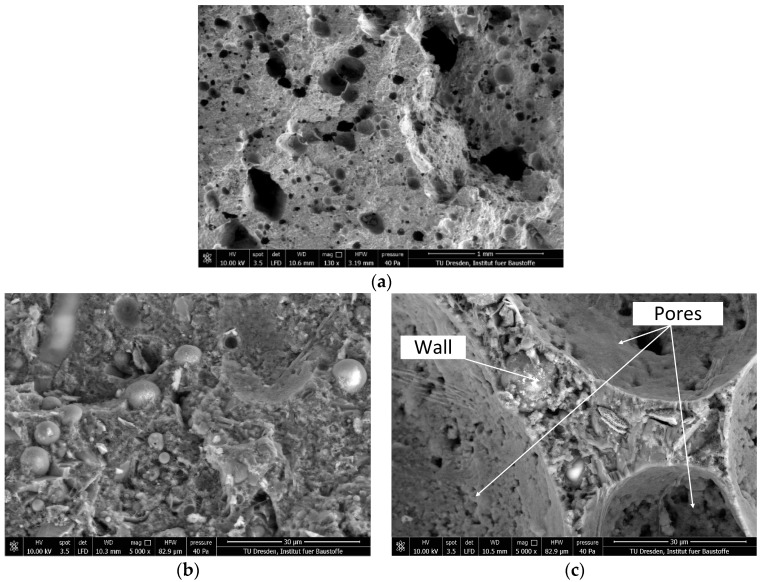
SEM pictures of the foam concrete M-2: (**a**) distribution and structure of pores, (**b**) matrix A-1 used for production of M-2, and (**c**) segment of the wall between pores.

Figure 11 shows exemplary and typical environmental scanning using an electron microscope (ESEM) images of the foam concrete M-2. The air bubbles have non-uniform geometry; their size is in the range of several tens to hundreds of micrometers; lastly, they are separated by a cement-based matrix, i.e., they form no continuous network; see Figure 11a. Jones et al. [7] suggested for foam concrete in general that spherical bubbles occurred due to entrapped air, and non-spherical bubbles were induced by the surfactant that created the foam. 

A higher magnification of matrix regions discloses that the microstructure is fairly dense and does not have significant capillary porosity, which would be in the range of pore diameters of some few up to tens of micrometers; see Figure 11b. This can be traced back to the relatively low water-to-binder ratio of composition A-1. Furthermore, a significant amount of unhydrated spherical fly ash particles is visible, which is typical of such a mixture composition type at a concrete age of 28 days. The fly ash is expected to undergo pozzolanic reactions over the course of time, with the hydration products densifying the matrix further. It may be concluded that neither the density of the microstructure nor the hydration characteristics of the fly ash were significantly influenced by foam addition or the 3D-printing process. 

The walls between the air voids constitute the load-carrying frame in the structure of the foam concrete. Figure 11c shows that these walls feature numerous very small, nearly spherical voids, some of which are located at the interface to the foam bubbles. Hence, the surfaces of the characteristic foam voids are not smooth. However, a dense matrix structure in conjunction with isolated large air voids, which stem from the foam added, are prerequisites for the high thermal insulation capacity, high compressive strength, and relatively low permeability of the foam concrete. Lastly, the very tiny voids that make the foam concrete interface non-coherent may impair the physical and mechanical properties of the hardened material. Further research is needed in order to be able to state the chemical reasons for the formation of these very little voids in the matrix and, as a next step, to avoid them. 

Interestingly, no cracks originated or terminated at the large-scale air bubbles that stem from the foam. This finding indicates that local tensile strains due to different drying shrinkage degrees were insignificant, which is in contrast to [7]. 

**Figure 12:**
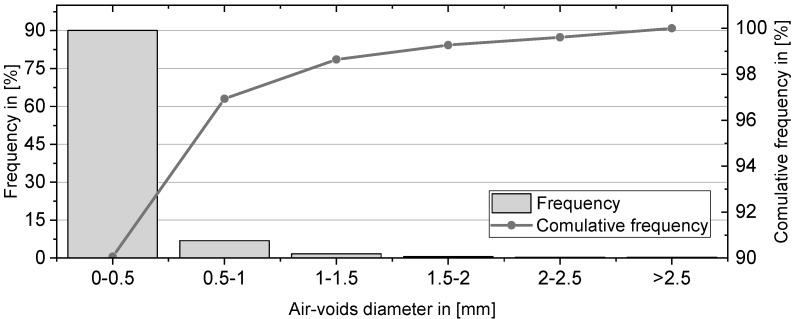
Air voids size distribution in the foam concrete M-2.

Figure 12 presents the air-void size distribution in the range of the deliberately introduced foam pores. The measured total porosity is 25.03%, and 90% of all the pores have a diameter smaller than 0.5 mm. The proportion of the pores between 0.5–1 mm in diameter is only 7%, while pores greater than 1 mm make up only 3% of the total number of pores. These findings obtained by digital light microscopy are in line with the more local observations by ESEM; see Figure 11. 

## 4. Conclusions

The potential application of foam concrete in digital construction was reviewed, and the mixture design for this novel material was developed and verified. The major findings of this work are as follows:

- Based on an established approach to the mixture design of self-compacting concrete, it was possible to manufacture 3D-printable foam concretes in the density rage of 970 to 1500 kg/m^3^. These concretes could be extruded and deposited layer-wise.
- The discrepancy between the designed target density and that achieved was in the range of 0.25% to 10%. Improvement in the stability of prefabricated foam, especially a reduction in the time-dependent drainage of water, should contribute to even more robust compositions in consecutive research steps.
- The water-to-powder ratio of 0.37 of cement-fly ash paste in a proportion of 60:40 by volume with the addition of 0.25% of the SP by weight yielded an eminently workable cement-based matrix which is appropriate for the intermixing of prefabricated foam with minimal brakeage of bubbles.
- Foam concrete composition M-2, with a dry density of 980 kg/m^3^, exhibited a compressive strength of 10.4 MPa after 28 days. Although these values are the results of laboratory-scale test specimens, they clearly indicate that printable foam concrete can prospectively be used for structural applications, e.g., load-bearing walls in multi-story houses. Further aspects such as testing the mechanical properties of real-scale prototypes need to be studied before the technology can be recommended for large-scale industrial implementation.
- From a broader perspective, walls with high thermal insulation (currently 0.24 W/mK) could be produced using additive technology directly on the site.

Future work should focus on rheological behavior by varying the foam contents and quantitative characterization of the possible anisotropy of the mechanical properties of printed foam concrete. Production techniques for continuous foam concrete production, including the mixed foaming method, should be considered as well. It is expected to achieve raw densities of printable foam concrete in the range of 800 to 900 kg/m^3^ with appropriate mechanical characteristics for structural applications.

## Figures and Tables

**Table 1:** Binder compositions tested following the Okamura procedure.

Binder	Cement Type	Composition by Volume [Fly Ash:Cement]	Fly Ash-to-Cement Ratio [by Weight]
A-0	CEM II	0:100	0.00
A-1	CEM II	40:60	0.47

**Table 2:** Chemical composition of cement and fly ash (LOI = loss on ignition, n.d. = not determined).

Material	Density [g/cm^3^]	Chemical Composition [% by mass]											
		Residue	SiO_2_	Al_2_O_3_	Fe_2_O_3_	CaO	MgO	SO_3_	K_2_O	Na_2_O	LOI	CO_2_	Cl
CEM II/A-M (S-LL) 52.5 R	3.12	0.74	20.63	5.35	2.82	60.94	2.14	3.52	1.05	0.22	3.47	2.87	0.07
Fly ash H4	2.22	n.d.	n.d.	n.d.	n.d.	3.6	n.d.	0.6	n.d.	2.9	1.8	n.d.	< 0.01

**Table 3:** Binder paste-mixing procedure for the determination of the powders’ water demand.

Time [min:s]	Speed [rpm]	Action
0:00	0	Add water to the solids
0:00–1:00	2500	Mixing at low speed
1:00–1:30	5000	Mixing at high speed
1:30–3:00	0	Rest, over this time, scrape the walls
3:00–4:00	5000	Mixing at high speed

**Table 4:** Foam concrete mixing procedure.

Time [min:s]	Speed [rpm]	Action
0:00	0	Add water to the solids in the mixing tank
0:00–2:00	3000	Mixing at high speed
2:00–2:30	0	Inspect the mixture for homogeneity
2:30–4:30	3000	Mixing at high speed
4:30–5:00	0	Adding 40% of the whole foam volume
5:00–7:00	1500	Mixing the matrix and the foam together at slow speed
7:00–8:00	0	Adding further 40% of the whole foam volume
8:00–10:00	1500	Mixing the matrix and the foam together at slow speed
10:00–11:00	0	Adding the remaining 20% of the whole foam volume
11:00–13:00	1500	Mixing the matrix and the foam together at slow speed

**Table 5:** Mixture compositions of the foam concretes under investigation. FC: foam concrete, SP: superplasticizer.

Mixture	Target Density [kg/m^3^]	Matrix (w/c)_eq_	FC (w/c)_eq_	Cement [vol% of FC]	Fly ash [vol% of FC]	Water [vol% of FC]	SP [wt% of binder]	Foam [vol% of FC]
M-1	1100	0.37	0.33	0.18	0.12	0.23	0.25	0.46
M-2	1200	0.37	0.34	0.20	0.13	0.25	0.25	0.41
M-3	1580	0.37	0.36	0.27	0.18	0.34	0.25	0.21

**Table 6:** Foam concrete properties in the fresh state.

Mixture	Matrix			Foam Concrete (FC)		
	Spread Diameter		Relative Spread * (matrix)	Spread Diameter		Relative Spread * FC
	Before Strokes [mm]	After Strokes [mm]		Before Strokes [mm]	After Strokes [mm]	
M-1	103	122	0.18	102	126	0.24
M-2	103	120	0.17	107	132	0.23
M-3	105	121	0.15	105	133	0.27

* Relative spread = before strokes/after strokes – 1.

**Table 7:** Overview of density characteristics of the foam concrete compositions under investigation.

Mixture	Target Fresh Density [kg/m^3^]	Fresh Density [kg/m^3^]	Deviation from Designed Density [%]	Dry Density [kg/m^3^]	Drying Weight Loss [%]
M-1	1100	1180	+6.78	970	17.8
M-2	1200	1197	−0.25	980	18.1
M-3	1580	1430	−10.5	1307	8.6

**Table 8:** Average mechanical properties of foam concrete M-2; standard deviations are given in parentheses.

Composition	Age [Days]	Flexural Strength f_ctm,fl_ [MPa]	Compressive Strength f_cm_ [MPa]
M-2	7	1.94 (0.05)	8.20 (1.01)
	28	2.12 (0.30)	10.40 (0.06)

**Table 9:** Results of the thermal conductivity measurements; standard deviation is given in parenthesis.

Composition	Average Density [kg/m^3^]	Average Value of λ [W/mK]	Source of the Values
M-2	980	0.24 (0.02)	study at hand
V-1	2100	1.68 (0.01)	study at hand
Concrete	2240	1.70	[55]
